# Polymeric Micelles for Breast Cancer Therapy: Recent Updates, Clinical Translation and Regulatory Considerations

**DOI:** 10.3390/pharmaceutics14091860

**Published:** 2022-09-03

**Authors:** Vijayabhaskarreddy Junnuthula, Praveen Kolimi, Dinesh Nyavanandi, Sunitha Sampathi, Lalitkumar K. Vora, Sathish Dyawanapelly

**Affiliations:** 1 Drug Research Program, Faculty of Pharmacy, University of Helsinki, Viikinkaari 5 E, 00790 Helsinki, Finland; 2 Department of Pharmaceutics and Drug Delivery, School of Pharmacy, The University of Mississippi, Oxford, MS 38677, USA; 3 Pharmaceutical Development Services, Thermo Fisher Scientific, Cincinnati, OH 45237, USA; 4 GITAM School of Pharmacy, GITAM Deemed to be University, Hyderabad 502329, India; 5 School of Pharmacy, Queen’s University Belfast, Belfast BT9 7BL, UK; 6 Department of Pharmaceutical Science and Technology, Institute of Chemical Technology, Mumbai 400019, India

**Keywords:** breast cancer, polymeric micelles, clinical translation, drug delivery, bioavailability, regulatory affairs

## Abstract

With the growing burden of cancer, parallel advancements in anticancer nanotechnological solutions have been witnessed. Among the different types of cancers, breast cancer accounts for approximately 25% and leads to 15% of deaths. Nanomedicine and its allied fields of material science have revolutionized the science of medicine in the 21st century. Novel treatments have paved the way for improved drug delivery systems that have better efficacy and reduced adverse effects. A variety of nanoformulations using lipids, polymers, inorganic, and peptide-based nanomedicines with various functionalities are being synthesized. Thus, elaborate knowledge of these intelligent nanomedicines for highly promising drug delivery systems is of prime importance. Polymeric micelles (PMs) are generally easy to prepare with good solubilization properties; hence, they appear to be an attractive alternative over the other nanosystems. Although an overall perspective of PM systems has been presented in recent reviews, a brief discussion has been provided on PMs for breast cancer. This review provides a discussion of the state-of-the-art PMs together with the most recent advances in this field. Furthermore, special emphasis is placed on regulatory guidelines, clinical translation potential, and future aspects of the use of PMs in breast cancer treatment. The recent developments in micelle formulations look promising, with regulatory guidelines that are now more clearly defined; hence, we anticipate early clinical translation in the near future.

## 1. Introduction

Breast cancer is one of the most frequently diagnosed cancers causing death among women, thereby posing a severe global burden [1]. Among the different types of cancers, breast cancer accounts for approximately 25% and approximately 15% of deaths. In 2020, nearly 2.3 million new cases of breast cancer were recorded worldwide along with 685,000 deaths caused by breast cancer, and the number of cases may rise to 4.4 million by 2070 [2]. As per the WHO report, nearly 7.8 million women were diagnosed and living with breast cancer in a period of five years (2015–2020) [3]. Breast cancer shows a greater loss of disability adjusted life years by women worldwide in comparison to any other cancer. Moreover, breast cancer was ranked first in the total incidence and number of deaths in most countries [2].

Molecular subtypes of breast cancer include human epidermal growth factor receptor-2 (HER-2), triple-negative breast cancer (TNBC), estrogen (ER)/progesterone receptor (PR)-positive or -negative molecular subtypes, and luminal A and luminal B [4] ER/PR-positive types of breast cancer are almost 70% of the cases usually reported. Among all the types of breast cancer, TNBC is the most aggressive and lacks the involvement of ER, PR, and HER-2 receptors, which are primarily responsible for the poor prognosis and diagnosis of the disease, resulting in elevated metastasis and drug resistance [4]. There are high chances of survival and recovery rates when breast cancer is detected at an early stage.

Normally, the adoption of any regime is based on the condition of the patient and the stage of the disease. The standard approach to treat breast cancer is to use cytotoxic chemotherapeutics before surgery, as it is a less invasive technology, or to reduce the extent of surgery, which is called neoadjuvant therapy [5,6]. Other regimens involve adjuvant therapy, where chemotherapy is given after the primary treatment to lower the risk of cancer recurrence. Since the 1970s, chemotherapies have been assisting oncology patients, including breast cancer patients. It is known that several anticancer drugs that are used clinically are mostly hydrophobic with poor solubility and bioavailability. The most commonly administered chemotherapeutics to treat breast cancer are paclitaxel (PTX), doxorubicin (DOX), epirubicin (EPB), docetaxel (DTX), cisplatin, carboplatin, etc. [7,8,9]. Furthermore, it was found that no single chemotherapy is good enough to treat cancer. Hence, several therapeutic regimens incorporate drug combinations, such as anthracycline–taxane combinations, which are used in the treatment of high-risk TNBC [4]. Similarly, sequential treatment in cases of moderate- to high-risk breast cancers as adjuvant therapies are available, which include EPB and cyclophosphamide following DTX or PTX treatment. Additionally, in neoadjuvant therapy, platinum-based drugs such as carboplatin, cisplatin, and anthracycline are used [10]. Several drugs are categorized as Biopharmaceutical Classification System (BCS) class II or IV with low solubility, permeability, and bioavailability and are cytotoxic [11]. Furthermore, many of the anticancer drugs show efficacy at higher doses, resulting in severe systemic side effects and less efficacy affecting normal healthy cells because of low specificity and sensitivity [12].

Nanotherapeutics are part of a powerful strategy employed to deliver hydrophobic drugs to overcome certain challenges and improve drug efficacy. Nanomedicine is a broadly applied term for various types of systems, such as liposomes [13], polymersomes [14,15], nanofibers [16,17], protein nanoparticles [18], nanosuspensions [19], emulsions [20], polymeric nanoparticles [21], and metallic nanoparticles [22], used in various exploratory or clinical studies [23,24]. Looking at the present scenarios, the fastest and most convenient solution to improve the cancer treatment regime is by designing new delivery systems that are easy to prepare, economic, have high drug loading efficiency, are stable under biological conditions, promote drug retention in tissues, and target delivery for already existing FDA-approved drugs. These characteristics can be harnessed with the use of polymeric micelles (PMs), the latest trending nanomedicines among the scientific community, showing high-quality structural characteristics [25]. Among other nanomedicines, PMs are self-forming agglomerates at the critical micellar concentration (CMC) of amphiphilic polymers [26]. This facile synthesis of nanostructures is advantageous for higher scale-up feasibility and the manipulation of loading capacity compared to other nanosized carriers, such as liposomal and polymeric nanoparticles, which demands costly, complex, and time-consuming manufacturing procedures [27,28]. Although an overall perspective of PM systems has been presented in recent reviews [29,30,31], a brief discussion has been provided on PMs for breast cancer. Therefore, the present review aims to provide an overall concept of PMs, recent updates on PMs, and various strategies for delivering cargoes to tumor sites in the treatment of breast cancer. In addition, we tried to shed light on the clinical translation potential and regulatory aspects of these systems.

## 2. Properties and Recent Updates of PMs

The most commonly employed hydrophilic part is polyethylene glycol (PEG), along with other emerging polymers, such as poly(vinylpyrrolidone) and poly(trimethylene carbonate) [32]. The hydrophobic components that are often used include poly(propylene oxide), polyesters, or copolymers of glycolic and lactic acids. Furthermore, the copolymer blocks are tunable to suit the end requirement by playing with combinations and ratios of hydrophobic/hydrophilic components. The size of PMs helps to improve the bioavailability of the drug, target-specific action, controlled release, and prolonged circulation time, thereby reducing drug-associated side effects. NanoPMs of a certain size (30 to 100 nm) can passively accumulate and reside within tumors since they are associated with abnormal vasculature, thereby producing desired results [32]. Furthermore, the long circulation of micelles can be attributed to surface characteristics; for instance, surfaces with a neutral and hydrophilic nature exhibit poor interaction with proteins, thus helping to improve the circulation time of micelles [33]. The administration of micelles through the oral route requires a positively charged surface, which helps to increase the penetration and mucoadhesive properties and enhance drug transport across biological barriers. However, positively charged surfaces are also associated with poor stability in biological environments due to nonspecific protein binding, which can lead to aggregation in vivo [34]. Therefore, a proper balance of charge on the surface of micelles is an important criterion for designing stable micelles [35]. With regard to improving the therapeutic effectiveness of the nanosystems, multiple drugs can be trapped within the micellar structure. Drug loading can be facilitated through physical entrapment or chemical conjugation with the micelle polymer. Mostly, drugs loaded using a physical entrapment procedure are released by easy diffusion, while chemically conjugated drugs are released by surface erosion or complete degradation of the PMs [36].

**Figure 1:**
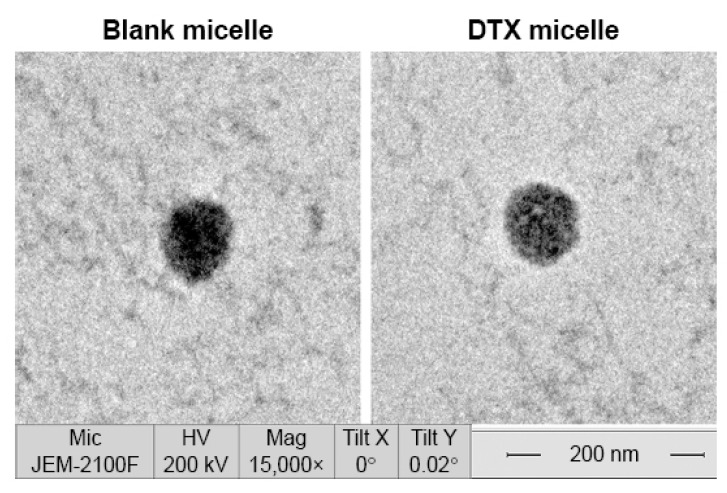
TEM image of blank micelles and doxorubicin (DTX)-loaded PM. The PM composition consists of poly(ethylene glycol)-poly(caprolactone) (PEG-PCL) (molecular weight: 3900 Da) with a two-block ratio of 1:1. Reproduced from [40] Originally published by and used with permission from Dove Medical Press Ltd.

The primary structural and physicochemical properties of the polymers can be determined by techniques such as nuclear magnetic resonance (NMR) and Fourier transform infrared spectroscopy (FTIR), which will help to understand the nature of amphiphilic components to help predict the property of the micellar system [35,37]. The CMC in plasma or other biological fluids is different than in water or buffer. Therefore, determining CMC in various solvent systems and storage conditions may be helpful to predict its stability. Determination of CMC can be carried out using methods that calculate surface tension or by assessing fluorescence intensity in solution or by dynamic light scattering [37]. A study investigated CMC using the conductivity meter of amphiphilic poly(ethylene glycols) (PEGs) as a hydrophilic block and aliphatic diacids, i.e., glutaric acid, adipic acid, pimelic acid, and suberic acid as hydrophobic blocks. The reported diameter of the PMs was in the range of 127.5–354 nm, and CMC values ranged from 112 to 155 mg L^−1^ [38]. Similarly, another study reported CMC values for amphiphilic blocopolymers composed of PEGs (hydrophilic block) and azelaic acid, sebacic acid, dimethyl isophthalate acid, and dimethyl terephthalate as hydrophobic blocks. The PM size ranged from 51.6–174 nm for aliphatic polymers, corresponding with CMC values between 95 and 130 mg L^−1^ and 135.5–371 nm for aromatic polymers, with CMC values ranging from 420 to 1500 mg L^−1^ for aromatic polymers [39]. The physical properties of PM can be determined using various experimental techniques, such as dynamic light scattering (DLS) and atomic force microscopy (AFM) and greater resolution techniques, such as cryo-transmission electron microscopy (cryo-TEM) and transmission electron microscopy (TEM) [32,35]. A summary is provided in Table 1. A representative TEM image of blank micelles and drug-loaded micelles 25–30 nm in diameter is shown in Figure 1 [40]. These techniques can also help to evaluate morphological changes in polymeric micelles induced by interactions with biological systems. Further advanced techniques, such as X-ray scattering, may be employed to understand the physicochemical properties of PM along with a better understanding of the structural organization of the outer and inner architecture of PMs loaded with drugs. Furthermore, X-ray scattering experiments can provide information on the behavior of PMs in the tissue microenvironment to predict biological responses. Another interesting technique is fluorescence resonance energy transfer (FRET), which aid in understanding the fluctuating nature of the active therapeutic agent in the core of the micelles [41]. Understanding this phenomenon helps to predict the stability of the PM structure and drug release profile under physiological conditions.

**Figure 2:**
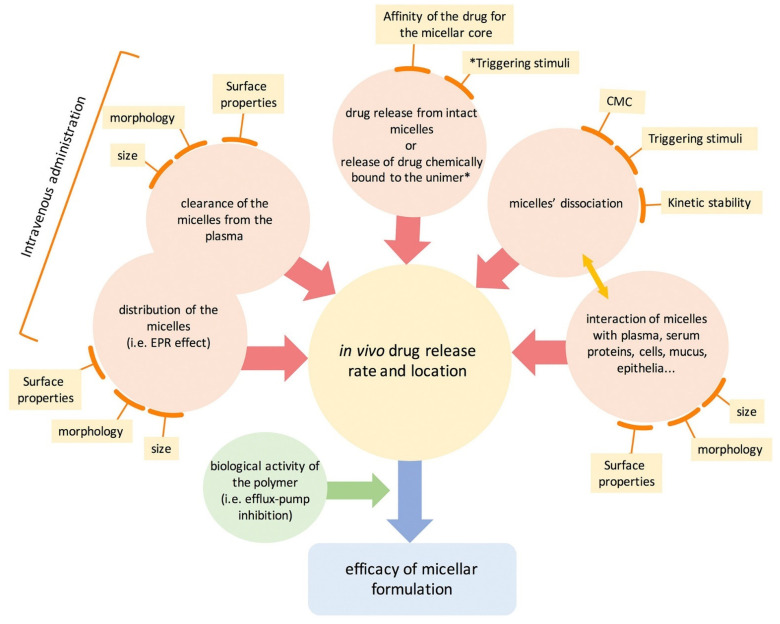
Schematic representation of the mechanisms of polymeric micelles. All mechanisms either work in a standalone manner or are mutually influenced by various conditions; unimer*, single unit of amphiphilic block; * Triggering stimuli represent various strategies, such as temperature, pH, enzymes, and magnetism. Note: EPR, enhanced permeability and retention; CMC, critical micellar concentration. Reprinted from [37] CC BY 4.0 License.

The position of the drug molecules within the PM is based on both the properties of the drug and the length of the polymer chain in the amphiphiles. Normally, a strongly hydrophobic drug is located within the PM core, which is hydrophobic, while drugs with intermediate polarity or high hydrophobicity will be present between the core and the surface or on the surface of the PM [32]. The interaction of drugs with the polymer may be due to simple electrostatic interactions or may arise due to covalent bonding. This results in different capacities of drug loading or encapsulation in the micelles [32,37]. As discussed, PMs are a simple assembly of amphiphiles at the CMC. This indicates that the structure formed is due to weak interactions and will fall apart when the CMC is altered, mostly upon dilution, thereby weakening the stability of the structure [37]. Therefore, the prime area of improvement is to design stable PMs that are stable enough upon administration in the bloodstream. Stable PMs will help prevent any premature leakage out of the encapsulated drugs, thus increasing the circulation half-life and helping to improve targeted drug delivery. Stabilizing PM can be achieved by cross-linking strategies, where strong bonds are created between adjacent polymers of the micelle system, which are intended to break only in response to stimuli [33]. Stimulus sensitization of the nanomicellar structure depends upon several external conditions, such as pH, redox, enzyme activity, hypoxia, light, and temperature [54]. By modulating their chemical structure, physicochemical properties, and stability under relevant conditions, nanomicelles can be enhanced to improve the target-specific delivery of drugs and control the rate of drug release in the tumor microenvironment. Several metallic nanoparticles act by inducing apoptosis and activating the mitochondrial membrane potential (MMP) [55,56,57]. However, upon exposure to stimuli, the PMs react by breaking open the micellar structure and consequently releasing the drugs. The release of drugs at the tumor-specific site minimizes off-target drug binding with minimum side effects (Figure 2).

**Figure 3:**
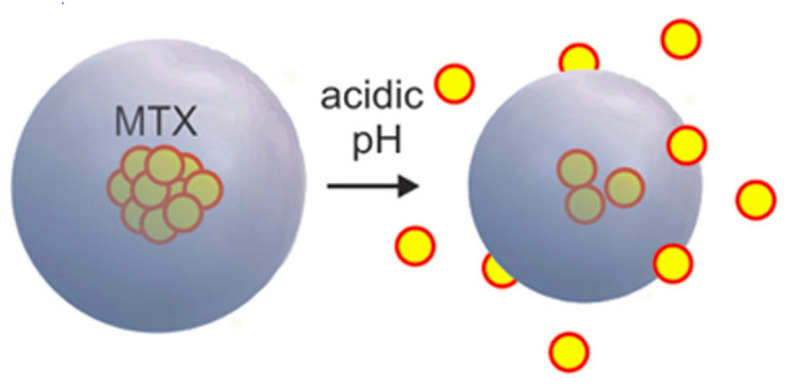
pH-sensitive micelles loaded with methotrexate (MTX) disassemble to release MTX in the presence of an acidic tumor microenvironment. A diblock copolymer of poly(monomethoxy ethylene glycol)-b-poly(ε-caprolactone) (mPEG-PCL) was used in the preparation, and the release was triggered by the sensitivity of the ester linkages to acidic pH. Reproduced from [60] CC-BY License.

pH-sensitive micelles that respond to the low pH stimuli of the tumor microenvironment are mostly used in multifunctional drug delivery systems [58,59]. One strategy for assigning pH sensitivity is the use of pH-based degradable linkers (acetals or hydrazones). These chemical groups form linkages between the hydrophobic and hydrophilic blocks of the PM. Micelles, when arriving at the tumor microenvironment with low pH, readily undergo degradation of the linkages between the hydrophobic and hydrophilic blocks, subsequently causing the disassembly of the PM and the release of drug molecules. Another strategy employs pH-responsive polymeric blocks such as poly(β-amino ester) and poly(amino acids), which will undergo charge conversion in response to a low pH stimulus, thereby causing alterations in the polymer structure and subsequent drug release (Figure 3). The reported CMC values for the pH-sensitive micelles were 18 µg mL^−1^ for a poly(monomethoxy ethylene glycol)-b-poly(ε-caprolactone) (mPEG-PCL) copolymer of 12,000 g mol^−1^ and 7.2 μg mL^−1^ for a copolymer of 7000 g mol^−1^.

Another property of the tumor environment that can be exploited is enzymes, which are characteristically overexpressed by tumor cells, such as, for example, in matrix metalloproteases (MMPs), which can degrade all kinds of extracellular matrix proteins. Furthermore, the activity of certain enzymes, such as NAD(P)H:quinone oxidoreductase-1 (NQO1), which are found in certain types of cancer microenvironments, can also be exploited. The enzymes are capable of catalyzing the depolymerization of PM constructs using enzyme-sensitive linkers or with the help of conjugated substrates [61].

**Figure 4:**
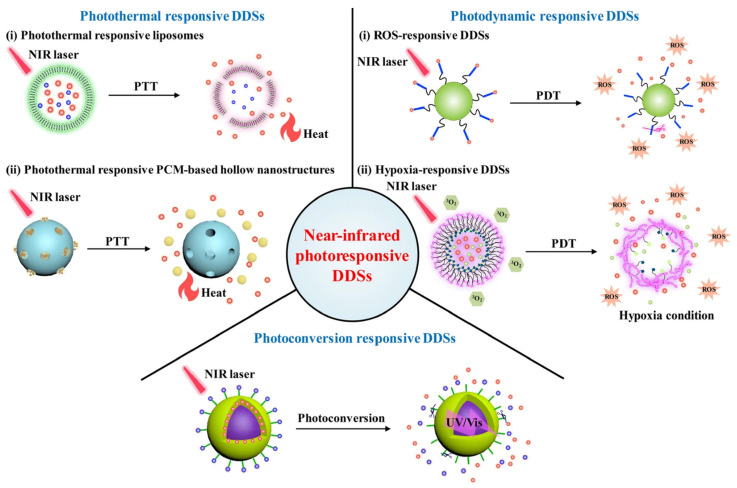
Temperature/photosensitive micelles entrapping drugs that are released in the presence of NIR. Note: drug delivery systems (DDSs); near-infrared (NIR), (650–950 nm); photothermal therapy (PTT); photodynamic therapy (PDT); reactive oxygen species (ROS); reproduced from [62] CC-BY License.

Elevated temperature in the tumor or an external stimulus inducing artificially elevated temperature with the help of temperature-sensitive agents can be exploited to release the drug on the trigger. Elevating temperature-based stimuli to promote drug release is simpler and easier without any further modifications. Temperature-sensitive polymers such as poly(N-isopropyl acrylamide) (pNIPAAm) and their derivatives are widely used to synthesize temperature-sensitive micelles. For the temperature-triggered release of drugs, an indirect method to induce a temperature rise can be performed by near-infrared (NIR) light-responsive micelles. A representative image of various photosensitive strategies is given in Figure 4. The micelles entrap NIR-responsive chemical entities or polymers together with drugs. The PMs under irradiation cause a rise in temperature, facilitating the disassembly of micellar structures and, consequently, the release of drugs. Many light-sensitive micelles are prepared using chemical moieties such as imidazoles, BODIPY, and 2-nitrobenzyl linkers [30].

**Figure 5:**
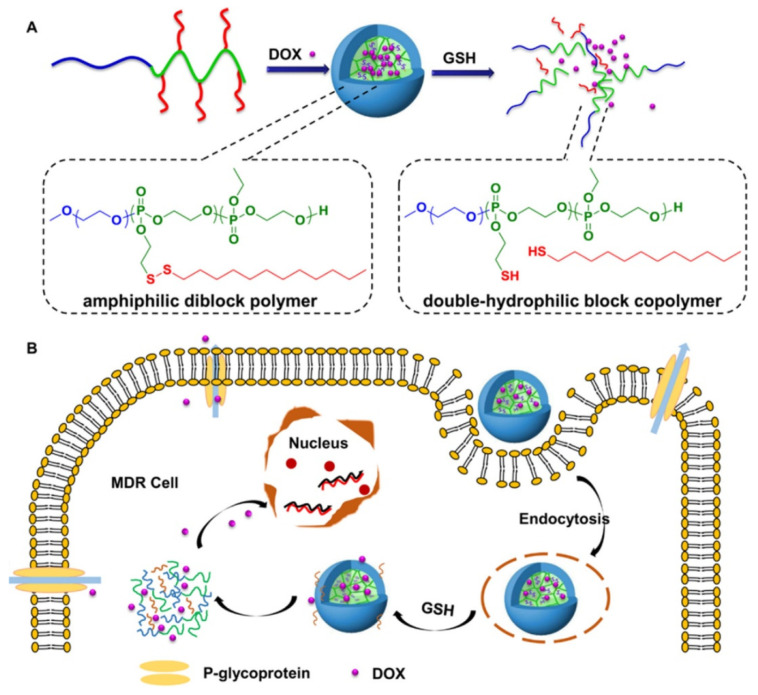
Schematic diagram showing the generation of redox-sensitive micelles made up of polyphosphoesters (PPEs): (**A**) mPEG_45_-*b*-P(DssEEP_12_-*co*-EEP_6_) and mPEG_45_-*b*-P(DssEEP_15_-*co*-EEP_20_). Note: N2-(2-(dodecyldisulfanyl)ethoxy)-1,3,2-dioxaphospholane 2-oxide (DssEEP). (**B**) Methoxy polyethylene glycol (mPEG) loaded with doxorubicin (DOX) is released in the presence of a GSH-rich milieu in the tumor microenvironment. Multidrug resistance (MDR), glutathione (GSH). Reproduced with permission from [64]. Copyright © 2015, American Chemical Society.

Another well-explored strategy involves developing reduction-sensitive PMs. Redox-responsive micelles respond to intracellular glutathione (GSH) by degrading the disulfide bonds present in the micelles, causing the disassembly of the micellar structures and release of entrapped drugs. The GSH concentration is higher in the cytoplasm of cells in comparison to plasma, and its concentration is four-fold greater in tumor tissues than in normal tissues [63]. The increased GSH levels in the tumor microenvironment cause the degradation of micelles, leading to the rapid delivery of therapeutic agents at the desired site (Figure 5).

Magneto-responsive micelles, which respond in the presence of a magnetic field, are yet another strategy. Such polymers are loaded with magnetic entities (iron, cobalt, or nickel species), which are chemically conjugated with the polymers or are embedded physically within the micellar structure [65]. The magnetic entities are present within the micelle in the presence of an alternating magnetic field; they align with the field and rotate, producing heat and thereby causing micellar-structure disruption and the release of the drugs [66].

## 3. PMs for Breast Cancer

Recent advancements made in PMs suggest that these systems can be designed to deliver the drug to targeted tissue and provide increased stability in the PM in biological conditions.

### 3.1. Conventional PMs

A combination of PTX and lapatinib (LAP) was entrapped within spherical micelles of polylactide-co-poly(ethylene glycol) (PLA-PEG). Drug release studies observed sustained delivery of LAP along with the release of PTX. The concentration of drugs in micellar solution and lyophilized micelles revealed a suitable storage capacity for a minimum of 6 weeks. The study revealed greater toxicity against breast cancer cell lines for PTX/LAP-entrapped micelles in comparison to the drug combination of PTX and LAP following 2 to 3 days of administration. This successful attempt also suggested the role of PTX/LAP micelles in the treatment of HER-2-negative along with HER-2-positive breast cancers [67]. Wan et al. developed a PM using a poly(2-methyl-2-oxazoline-*block*-2-butyl-2-oxazoline-*block*-2-methyl-2-oxazoline) (P(MeOx-*b*-BuOx-*b*-MeOx) amphiphilic block copolymer trapped inside PTX and alkylated cisplatin drugs. Studies revealed multiple advantages of this PM system, including the gradual release of the drugs in the serum, better drug distribution across the tumor microenvironment, and improved pharmacokinetics for both drugs. In vivo experiments using PTX/CP PMs in A2780/CisR xenograft tumors (cisplatin-resistant human ovarian carcinoma) and LCC-6-MDR (multidrug-resistant breast cancer) orthotopic tumor models showed enhanced antitumor activity. A 3D model simulation of the PM in a serum with tumor compartments revealed that the superior antitumor activity was due to the slower rate of drug release by the micelles. This drug delivery system loaded with PTX and cisplatin showed favorable results, thereby highlighting their clinical potential in the treatment of breast and ovarian cancer [68].

Another example demonstrates the encapsulation of PTX in the PM of poly(ethylene glycol)-*block*-dendritic polylysine. PTX is bound to the core comprising phenyl and thiourea groups via *π–π* stacking and hydrogen bonding interactions. This PM (PEG-G3-PEITC/PTX) was reported to have better release, slower clearance, and tumor accumulation properties than micelles in clinical use, such as PTX-loaded poly(ethylene glycol)-*block*-poly(*D*,*L*-lactide) (PEG-PDLLA/PTX). PEG-G3-PEITC/PTX showed enhanced therapeutic efficacy in both orthotopic human breast cancer and subcutaneous breast cancer xenografts, thereby suggesting a promising strategy for treating breast cancer [69]. A PM (SP1049C) loaded with DOX was synthesized to evaluate its efficacy against cancer stem cells (CSCs) in TNBC. SP1049C showed many advantages over free DOX, such as impeding the action of breast cancer resistance protein (BRCP) in CSCs, greater cytotoxicity against CSCs, and greater antitumor efficacy in orthotopic animal tumor models [70]. Liu et al. reported the synthesis of biodegradable PM with polyethylene glycol, polyglutamic acid, and polyphenylalanine (PEG-PGlu-PPhA) entrapped with DOX containing bone-homing alendronate (ALN) for the treatment of breast cancer metastasis to bone. These micelles had a bone-protecting capacity along with enhanced toxicity against tumor cells, sustained release, and improved pharmacokinetics. An in vivo experimental study involving syngeneic animal models with late-stage breast cancer bone metastasis showed reduced tumorigenesis and enhanced animal life span, supporting the potency of this osteotropic nanomedicine for the treatment of breast cancer metastasis to bone [71].

Sabra et al. reported the synthesis of self-assembled nanocarriers using hydrophobic zein with hydrophilic lactoferrin (Lf) protein to entrap rapamycin (RAP) and wogonin (WOG) drugs. This combination of the system improves the circulation time and tumor-specific targeting of drugs. Furthermore, they also reported crosslinking by glutaraldehyde, which resulted in reduced particle size and improved micellar stability. The nanocarriers demonstrated a fast release of WOG, which inhibits the efflux pump, thereby sensitizing the tumor cells to RAP, which is slowly released by PM. The brush-like architecture of the Lf shell resulted in better hemocompatibility together with serum stability. The synergistic combination of the two drugs showed improved cytotoxicity in the breast cancer cell line and improved tumor site targeting in in vivo experimental studies using tumor-induced animal models (Ehrlich ascites carcinoma [72]. In another study, gambogic acid (GA) with good antitumor potential was encapsulated within a PM made of monomethyl poly(ethylene glycol)-poly(caprolactone) (MPEG-PCL) copolymer. GA is highly hydrophobic, and the free drug shows much lower antitumor activity. GA-encapsulated nanocarriers showed increased aqueous dispersity, greater cytotoxicity and apoptosis of tumor cells, and better uptake into the tumor microenvironment in a breast cancer cell line (MCF-7) and in an MCF-7 subcutaneous xenograft tumor model [73].

Apart from synthetic drug molecules, naturally occurring molecules such as resveratrol (RES) have shown potent biological activity. However, its clinical uses are limited due to poor aqueous solubility; hence, Gregoriou et al. developed Res-loaded nanoparticles using Pluronic F127 block copolymer along with Vitamin E-TPGS for the entrapment of highly hydrophobic drugs. The nanoparticles also demonstrated the killing of target-specific breast cancer cells along with the potential to treat mammary malignancies [74]. Another study by Gener et al. demonstrated the efficacy of Zileuton^TM^ as a potent inhibitor of CSCs of breast cancer, originally approved by the FDA as an anti-leukotriene oral drug treatment for asthma. Encapsulated Zileuton^TM^ reduced the percentage of CSCs present in the tumor and efficiently hindered the circulating tumor cells (CTCs) in blood circulation, thereby preventing its metastatic spread [75]. A PM system of polyion complex (PIC) micelles was developed to encapsulate estrone, a type of estrogen that binds to estrogen receptors (ERα), which are usually overexpressed in the case of hormone-responsive breast cancers. Here, estrone was conjugated to poly(ethylene glycol) methyl ether methacrylate-b-poly tert-butyl methacrylate (POEGMEMA-PtBuMA) along with melittin to form PIC micelles. The estrone-containing PIC micelles showed enhanced cytotoxicity in 2D and 3D models of MCF-7 breast cancer cells. POEGMEMA-PtBuMA showed improved cellular uptake by tumor cells, aiding in targeted therapy. Further stability of PMs was improved by cross-linking of the PIC micelles, which also prevented melittin degradation from enzymatic attack. These observations suggest the therapeutic role of these nanocarriers in targeted drug administration and superior antitumor activity against hormone-responsive breast cancer cells [76].

The natural flavonoid fisetin (FS), being hydrophobic, was encapsulated in pluronic127 (PF)-folic acid (FA)-conjugated micelles (FS-PF-FA) to improve its bioavailability and targeted delivery. FA-conjugated PF was synthesized by chemical crosslinking using carbodiimide, which was further used to prepare FS-PF-FA micelles using the thin-film hydration technique. FS-PF-FA bioavailability increased six-fold with no toxicity, greater systemic circulation, and slower plasma elimination compared to free FS. These micelles showed active tumor-specific targeting along with greater growth inhibitory effects against breast cancer cells (MCF-7) compared to free drug or FS-loaded PF micelles [77].

Kesharwani et al. developed self-assembling micellar structures of the plant-based polymer inulin (Inutec-SP1^®^, INT). These nanocarriers were loaded with a combination of DOX and/or PTX to study the antitumor effects in breast cancer. The INT-formed micelles had superior drug loading efficiency with sustained drug release. The micelles entered tumor cells through the process of endocytosis (clathrin-mediated), which was then transported to lysosomes, thereby causing the release of drugs within the tumor cell. This system, with a combination of DOX and PTX, showed enhanced antitumor potential compared with any other system. Moreover, these micelles had a greater in vivo circulation time along with targeted delivery at the tumor site. This unique system demonstrates a promising strategy for treating breast cancer more efficiently than free drugs [78].

**Figure 6:**
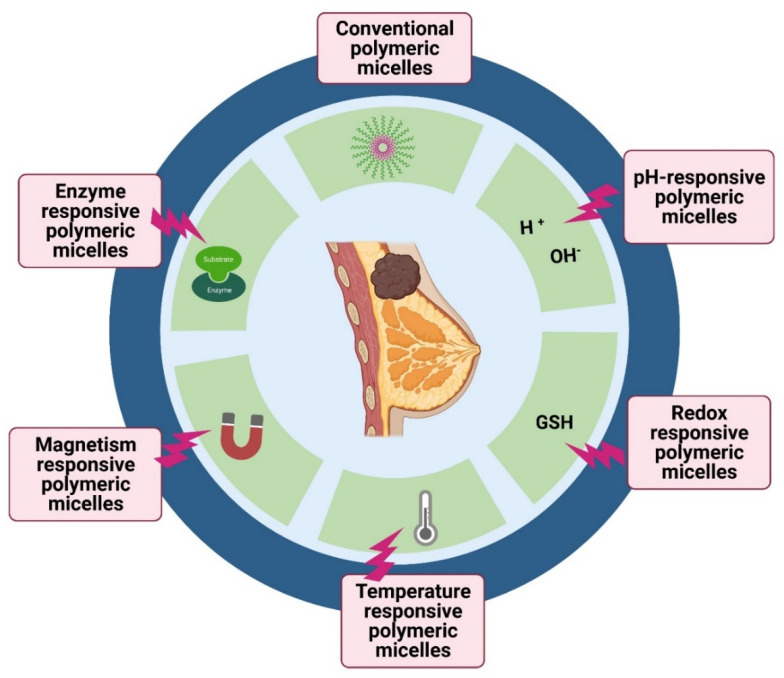
Various strategies in micellar tumor targeting to deliver therapeutic cargoes.

Another study demonstrated the delivery of DOX along with a unique biologically modified therapeutic miRNA prodrug (tRNA-mir-34a). The multifunctional nano micellar carrier was constructed by conjugating the amphiphilic copolymer POEG-VBC backbone with creatine (POEG-PCre). The system exhibited effective synergism in antimetastatic and activity both in cellular and animal models [79]. The solubility of poorly soluble Chrysin (CH), a phytoconstituent with anticancer activity, was enhanced by loading mixed micelles comprising pluronic PF127-pluronic F68 conjugated with folate, which enhanced the bioavailability and anticancer properties of CH in the MCF-7 breast cancer cell line by its target-specific action [80]. Figure 6 summarizes various strategies in micellar tumor targeting to deliver therapeutic cargoes.

### 3.2. pH-Responsive PMs

The P-glycoprotein (P-gp) transporter pumps out DOX, leading to a lower concentration of drug within the cell and inducing multidrug resistance (MDR). Thus, to overcome this issue, a multifunctional strategy was developed by Cao et al., where the chemotherapeutic drug was delivered by hyaluronic acid-deoxycholic acid-histidine together with a Pluronic F127-containing micellar system (HA-DOCA-His-PF). HA-DOCA-His-PF was internalized into tumor cells through endocytosis mediated by the CD44 receptor. The micelle disintegrated in response to the endosomal pH (5.5), releasing DOX within the tumor cell. This multifunctional drug delivery system showed superior antitumor activity toward a drug-resistant breast cancer cell line (MCF-7/Adr), confirming its potential for reversing MDR in breast cancer [81].

pH stimuli-responsive, core-shell nanofibers were incorporated with cholic acid conjugated with poly(bis(carboxyphenoxy)phosphazene PMs encapsulating the PTX drug. In vitro studies revealed a sustained release of the drug in the acidic tumor microenvironment along with good biocompatibility. These drug-loaded nanofibers showed greater cytotoxic potential than free PTX in breast cancer cells. The results confirm the promising targeted action and enhanced antitumor activity of the nanofibers, which have great potential in the safe and effective treatment of breast cancer cells [82].

An amphiphilic pH-responsive micelle structure consisting of copolymer, poly ((1,4-butanediol)-diacrylate-b-N,N-di-isopropylethylenediamine)-polyethyleneimine (BD-PEI), along with a matrix metalloproteinase (MMP)-responsive polymer, poly((1,4-butanediol)-diacrylate-b-N,N-di-isopropyllethylenediamine)-peptide-polyethylene glycol (BD-peptide-PEG) loaded with DTX, was prepared (pDM). The PEG group in pDM is lysed by MMPs in the tumor microenvironment, making the micelle more positively charged and smaller in size, facilitating passive internalization into tumor cells by the enhanced permeability and retention (EPR) effect and delivering increased amounts of the drug DTX in response to the acidic pH in the tumor site. Therefore, pDM micelles with dual MMP/pH sensitivity showed increased systemic circulation, targeted delivery, rapid uptake by tumor cells, and immediate drug release, serving as an effective platform for targeted drug delivery in metastatic breast cancer [83]. To improve the therapeutic potential of EPB, an anthracycline drug, pH-sensitive PMs loaded with EPI were produced (EPI/m) for the treatment of axillary lymph node metastasis (ALNM) of TNBC. The intratumoral acidic environment of ALNM prompted the selective accumulation of the drug along with its activation in these tumors, efficiently suppressing the growth of primary tumors and ALNM. These PMs could improve the therapeutic window of the active drug in a dose-dependent manner, thereby contributing to greater antitumor activity [84].

Two drugs, PTX and honokiol (HNK), were entrapped into micelles made of pH-responsive polymers, poly(2-ethyl-2-oxazoline)-poly(D,L-lactide) (PEOz-PLA), to suppress both MDR and breast cancer metastasis. This system improved cytotoxicity and uptake by drug-resistant breast cancer cells (MCF-7/ADR). Possible mechanisms of MDR reversal and the inhibition of metastasis include the downregulation of P-glycoprotein (P-gp) expression, increased plasma membrane fluidity, pH-triggered drug release, and PTX/HNK-based MMP inhibition, thus explaining the promising role of PTX/HNK codelivery using pH-responsive micelles for the treatment of MDR-resistant and metastatic breast cancer [85]. A pH-sensitive micellar cargo made up of poly(ethylene glycol)-b-poly(D,L-lactide)-b-poly(β-amino ester) (PELA-PBAE)-entrapped PTX was synthesized by Wang et al. to enhance the inhibitory action of PTX on metastatic breast cancer. This micellar system showed enhanced intracellular uptake and increased cytotoxicity by inducing apoptosis of tumor cells along with the inhibition of breast cancer metastatic cells (4T1) [86]. Another pH-sensitive PM was developed by Han et al. to improve the delivery of PTX specifically in tumor-bearing regions. This nanocarrier was synthesized using an amphiphilic conjugate containing mPEG and hydrazone-bonded, cholesterol-modified chitosan (mPEG-CS-Hz-CH). mPEG-CS-Hz-CH loaded with PTX showed spontaneous discharge of the drug into the tumor microenvironment with a pH of ~5. In vitro studies demonstrated no toxicity of the polymer conjugate along with significantly greater and selective cytotoxicity toward tumor cells. Experimental in vivo studies clearly validated the superiority of this PM, which not only reduced the side effects caused by PTX, but also improved its therapeutic efficacy [87].

Palanikumar et al. developed a novel strategic nanoparticle with a polylactic-coglycolic acid (PLGA) core crosslinked covalently with a bovine serum albumin (BSA) shell that decreased the interactions with macrophages and serum proteins, preventing recognition of the target [88]. The BSA shell is further functionalized with an acidity triggered rational membrane (ATRAM) peptide to target the tumor-specific microenvironment. The acidic nature of the tumor microenvironment disintegrated the nanoparticle, facilitating the release of the doxorubicin-triphenylphosphine (DOX-TPP) drug into cancer cells. Cellular and in vivo experiments in cancer cells and mice revealed greater cytotoxicity toward cancer cells (selectively breast cancer), with no toxicity toward normal healthy cells [89]. Another drug delivery system for DOX was developed by Song et al., who employed poly(ethylene glycol) methyl ether-D_labile_-poly(caprolactone)-ferrocene (mPEG-D_labile_-PCL-Fc) to synthesize amphiphilic block copolymer-based micelles with pH-responsive drug release. This carrier showed good biocompatibility together with good tumor-targeted delivery. The drug-loaded nanomicelles showed improved anticancer effects, as observed in in vitro experimental studies. The presence of ferrocene in the copolymer presented additional anticancer effects of increased ROS production in the tumor microenvironment [90]. In an attempt to improve the delivery of methotrexate (MTX) in a sustained manner within the tumor microenvironment, a group of researchers from Mexico developed diblock copolymer micelles of poly(monomethoxy ethylene glycol)-*b*-poly(ε-caprolactone) (mPEG-PCL) loaded with MTX. These PMs were pH-sensitive and promoted the release of drugs within the tumor microenvironment, which is associated with low pH. In vitro, an experimental study on MCF-7 breast cancer cells revealed a gradual release for over 22 days with a good inhibitory concentration, thus exhibiting a promising drug delivery system for breast cancer treatment [60].

### 3.3. Redox-Responsive PMs

Gulfam et al. synthesized a biocompatible block copolymer based on methoxy poly(ethyleneglycol)-b-poly(ε-caprolactone-co-α-azido-ε-caprolactone)(mPEG-b-poly(εCL-co-αN3εCL)) with reduction-sensitive bis(alkyne) crosslinking loaded with the methotrexate (MTX) drug with antitumor activity. The experimental studies revealed that crosslinked polymers loaded with MTX have controlled drug release and greater apoptotic potential against breast cancer cell lines (MCF-7) in comparison to noncrosslinked micelle and free MTX [91]. Self-assembling, redox-sensitive PMs were developed for the delivery of DOX. The PMs also contained hyaluronic acid backbone-bound ibuprofen (BF) linked through a disulfide bond (HA-ss-BF), which is redox-sensitive. In the tumor microenvironment, excess GSH triggers the breakage of the disulfide bond and disassembly of micelles, leading to the release of the drug and BF (anti-inflammatory drug). In vivo studies in breast cancer mouse models revealed suitable cellular uptake by tumor cells and good biodistribution [92]. An efficient drug/gene codelivery of a human tumor necrosis factor-related, apoptosis-inducing ligand (pORF-hTRAIL) and DOX was demonstrated by redox-sensitive amphiphilic PMs made of T7-conjugated polyethylene glycol-polyethyleneimine-poly(caprolactone)-S–S-poly(caprolactone)-polyethyleneimine-polyethylene glycol (PEG-PEI-PCL-S–S-PCL-PEG) (PPPT) for the codelivery of pORF-hTRAIL and DOX in the treatment of breast cancer. These micelles had good biocompatibility with no hemolytic toxicity, blood clotting, or activation of the complement system. In vitro experiments in MCF-7 cells and in vivo studies in breast cancer in a mouse model revealed greater cellular uptake efficiency and tumor accumulation, thus suggesting a promising codelivery platform for the treatment of breast cancer [93].

Chemotherapy is one of the main reasons for failure in anticancer treatment because of the inadequate delivery of drugs in the affected tumor microenvironment. Thus, to overcome this issue, a diblock copolymer was developed comprising PEG and polyphosphoester (PPE) as the hydrophilic and hydrophobic groups, respectively, and PPE containing disulfide bond-linked side chains loaded with DOX. The high glutathione levels in the drug-resistant breast cancer cells prompted the cleavage of disulfide bonds in the micelle and the rapid release of the drug with enhanced tumor cell death. The in vivo profile in a multidrug-resistant breast cancer mouse model showed a significant reduction in tumor volume, explaining the potential of this drug delivery system (copolymer micelle) for the clinical treatment of cancers involving drug resistance [64].

The solubility of cancer drugs and their off-target toxicity led to the design and development of block copolymer micelles, with disulfide bonds comprising a hydrophilic PEG group and a hydrophobic polycaprolactone group along with an active targeting RGD (arginine-glycine-aspartic acid) peptide group for targeted delivery in response to the high glutathione content in the tumor microenvironment. DOX and ICG (indocyanine green) were encapsulated within the redox-responsive nanomicelle to study the anticancer effects of chemotherapy along with photothermal therapy (PTT) in breast cancer. The in vivo experiments in breast cancer mouse models revealed a greater anticancer efficacy, improved solubility of both drugs, and stability of ICG, thus highlighting the superiority of this delivery system in the treatment of breast cancer [94].

Another redox-responsive micelle was developed by a group of researchers for the enhanced activity and target-oriented delivery of camptothecin using poly(tyrosine(alkynyl)-OCA) and monomethoxypoly(ethylene glycol)-b-poly(tyrosine(alkynyl)-OCA). The PMs contained a disulfide bond-based core cross-linking that promoted the redox-responsive disruption of the micelle for rapid drug release. These micelles showed exceptional stability under physiological conditions and rapid disruption in response to redox conditions with improved efficacy when used to treat breast cancer cells [95]. Bin et al. developed a redox-responsive, star-shaped PEG-sheddable copolymer of disulfide-linked PEG-lysine-di-tocopherol succinate (P_5k_SSLV) encapsulating DOX to study its inhibitory mechanism in an MDR breast cancer cell line (MCF-7/Adr). The P_5k_LV-DOX micelles showed the significant accumulation of DOX in the nucleus accompanied by increased apoptosis in MCF-7/Adr cells, thus conveying the potential of this micelle as a promising drug delivery system in drug-resistant breast cancer scenarios [96].

### 3.4. Temperature-Responsive PMs

He et al. [97] reported the use of PMs using a 1,2-distearoyl-sn-glycero-3-phosphoethanolamine-N-methoxy (polyethylene glycol)-2000 (DSPE-PEG2000) copolymer loaded with IR780 (IPM), which is a near-infrared sensitive dye used for various applications, such as cancer PTT, photoacoustic imaging (PAI), and fluorescence imaging (FI). These micelles were developed with the aim of PTT against lymphatic breast cancer metastasis. Experimental results revealed that under, 808-nm laser irradiation, the IPMs generated excessive heat, thereby killing tumor cells. IPMs specifically accumulated in the tumor sites and metastatic lymph nodes on intravenous administration. Based on the derived results, it was observed that a single IP treatment, together with laser irradiation, greatly reduced the growth of the primary tumor and inhibited lymphatic metastasis by 88.2% [97].

Another example demonstrated loading of IR780 along with ⍺-lipoic acid and DTX in the mPEG-PCL copolymer. ⍺-Lipoic acid improved the drug entrapment efficiency and strength of micelles to IR780 dye while maintaining the loading capacity of DTX. These micelles showed no hemolytic toxicity and were successfully used as FI/PAI probes for imaging together with PTT. In vitro and in vivo evaluation in breast cancer cells and mouse models demonstrated that the DTX/IR780 coloaded micelles had better antitumor potential than other micellar formulations or free forms of drug and dye [98]. Shikonin, a traditional Chinese drug, is known for its anticancer activity due to its potential to bind to multiple targets in tumor cells. However, its low solubility and delivery at the target site remain a major challenge; thus, Su et al. developed a thermosensitive nanomicelle comprising poly(N-isopropylacrymaide) and polylactic acid blocks with variable lengths. Shikonin was encapsulated within this copolymer micelle for its sustained and targeted delivery within the tumor site. In vitro studies in breast cancer cells revealed greater cytotoxicity of the drug compared with the free drug. In vivo studies in mice bearing breast cancer showed enhanced drug efficacy due to increased accumulation in the drug within the tumor microenvironment as a result of the higher temperature in the tumor site to volume phase transition temperature, causing the release of the drug [99].

### 3.5. Magnetism-Responsive PMs

Magnetic PMs were prepared using a hydrophobic oleic acid-coated magnetite (Fe_3_O_4_) core with an amphiphilic copolymer of zein-lactoferrin encapsulated with dasatinib (DAS). These micelles showed hemocompatibility and serum stability with controlled release of DAS in the acidic pH of the tumor microenvironment. In the presence of a magnetic field, these micelles expressed approximately 1.35-fold greater cytotoxicity against the TNBC cell line (MDA-MB-231) than micelles administered in the absence of a magnetic field. Under the effect of the magnetic field, the PM showed targeted delivery as a result of lactoferrin corona, greater inhibition of cellular migration, along with the inhibition of phosphorylated c-Src (target oncogene for TNBC) [52].

Varshosaz et al. developed magnetic iron oxide nanoparticles (MNPs) comprising an amphiphilic copolymer of folate-conjugated dextran/retinoic acid coated with oleic acid. These MNPs were synthesized for the controlled release of DOX for the treatment of breast cancer. In vitro cytotoxicity studies showed an improved inhibitory concentration of DOX in two kinds of breast cancer cell lines, including less binding affinity to serum proteins that affect its targeted delivery [65]. Song et al. developed a supersensitive ferrimagnetic micelle with ferrimagnetic polyethylene glycol-poly(2-hexoxy-2-oxo-1,3,2-dioxaphospholane) (mPEG-b-PHEP) encapsulating emodin drug and iron oxide nanocubes, which are hydrophobic with greater heating conversion efficiency. This micelle exhibited magnet-based targeting, a quick response to magnetic hyperthermia promoting supersensitive drug release upon alternating magnetic field (AMF) activation and enhanced antitumor potential against breast cancer cells, as observed in experimental studies [66].

Sang et al. [100] synthesized dual redox/pH and magnetically responsive PMs for the delivery of gambogic acid (GA), a potent anticancer agent with very low biocompatibility. The PMs comprise hexadecanol-modified chitosan oligosaccharide PMs (CSO-SS-Hex), which are reducible and coated with hyaluronic acid (HA) along with deoxycholic acid grafted PEG-PLL (sPEG) encapsulating GA and Fe_3_O_4_ nanoparticles. The in vitro and in vivo studies of this intelligent delivery system with pH and magnetic responsive functionality revealed rapid drug release at the tumor site with improved drug uptake in the tumor cells, explaining the potential of this PM in the treatment of TNBC [100]. Among the different release mechanisms, redox, enzyme, and pH strategies are widely used in most cancers for easy triggering, while thermal (IR) and magnetic strategies are sometimes limited to certain cancers, as their allowance depends on the locations of the tumor. This observation is true in the case of breast cancer treatments, despite several studies reporting in the literature that this strategy is not available in any clinical phase.

### 3.6. Enzyme-Responsive PMs

Han et al. developed an enzyme-responsive nanomicelle using amphiphilic polypeptides with the sequence stearyl-HHHRRRRR (SHR) along with a maleimide-modified, MMP-sensitive sequence (PLGLAGK-Mal) loaded with CH223191, an AhR inhibitor, and coated with an anti-CD28 antibody. These MMP-sensitive micelles enter the tumor microenvironment as a result of enhanced permeability and retention effects, where they are disrupted due to an increased MMP-2 enzyme in the tumor site, thus releasing the antibody and the inhibitor in a controlled manner. The potency of these novel micelles was studied in vitro and in vivo using breast cancer cell lines and breast cancer mouse models. These studies revealed very low cytotoxicity, good cellular uptake efficiency, and an enhanced immune response against breast cancer by modulating the tumor immune microenvironment [101].

A dual enzyme and pH-responsive micelle were synthesized by Luo et al. to overcome the challenging issue of the lung metastasis of breast cancer. The cathepsin B and pH-responsive block copolymer (hydroxypropyl methacrylate (HPMA) and oligo-(ethylene glycol) methacrylate (OEGMA)) and enzyme-sensitive peptide (GFLG) micelle were conjugated with DOX and loaded with nifuroxazide. Cathepsin B is overexpressed in the tumor microenvironment, which triggers the release of the drugs, aiding in the targeted delivery of the drugs in a controlled manner. These micelles showed enhanced antitumor activity with increased apoptosis and effectively inhibited metastasis in breast cancer mouse models, thus proving to be a potent nanomedicine against breast metastasis [102]. The different types of PMs developed against breast cancer, including polymers and therapeutic agents, are listed in Table 2.

## 4. Clinical Studies

Looking at recent advances in nanomedicine, they appear promising candidates for clinical translation. Since the beginning of the development of nanomedicines, 58 nanoformulations in the form of imaging and therapeutic agents have been permitted for clinical use [103]. The approved nanoformulations are primarily made up of liposomes, polymers, and inorganic materials for various indications. Approval of nanotherapeutics for clinical use is based on the therapeutic efficacy and safety profile. Thus, for its medical use, the safety and efficacy assessment of new therapeutics is conducted through clinical trials. Any novel therapeutic invention undergoes clinical trials consisting of three main phases, and, when successful, the therapeutic invention may receive approval for commercial use. The prime phases include Phase I, in which the effects of the medication on healthy human participants at the highest dose can be administered to determine the dose size that can be tolerated and to understand the pharmacokinetic profile; Phase II trials provide more information about any side effects and are performed on a large number of human participants who have underlying disease conditions that the new therapy is meant to treat; Phase III involves the monitoring of safety, efficacy, and dosage of a larger number of human participants who have the condition for the new therapy.

Presently, PMs, which are under investigation, are based on the delivery of potent but poorly water-soluble drugs for different cancer treatments. Particularly for breast cancer-related treatment, a few micelle systems have entered different phases of clinical trials [104]. Some of the PMs that have been clinically approved or are undergoing clinical trials for determining their safety and efficacy in various cancers, including breast cancer, are given below (Table 3).

PTX is one of several drugs that is used to treat various cancer indications. However, to improve its efficacy, PTX in nanoform was developed as Genexol-PM by loading PTX in PEG-b-PLA-based PMs. The size of these micelles was reported to be 25 nm, and they are undergoing clinical trials for various cancer types, including breast cancer. They have shown favorable efficacy in patients for the treatment of breast cancer [105]. Another polymeric technology called NK105 is a preparation of PTX loaded in PEG-b-PPBA (poly (4-phenyl-1-butanoate-L-aspartamide)) PMs with an average size of 85 nm, which also showed potent activity against breast cancer [106]. Another drug candidate, DTX, is widely employed to develop a micellar system such as Nanoxel-PM [107]. This system is made by a DTX-loaded PEG-PDLLA micellar formulation with a 25 nm diameter that has entered clinical trials. Another PM of DTX is Docecal by Oasmia Pharmaceutical, which was developed by combining DTX with N-all-trans retinoyl cysteine methyl ester sodium salt and N-13-cis retinoyl cysteine methyl ester sodium salt (XR-17) block polymer for breast cancer treatment [104].

**Figure 7:**
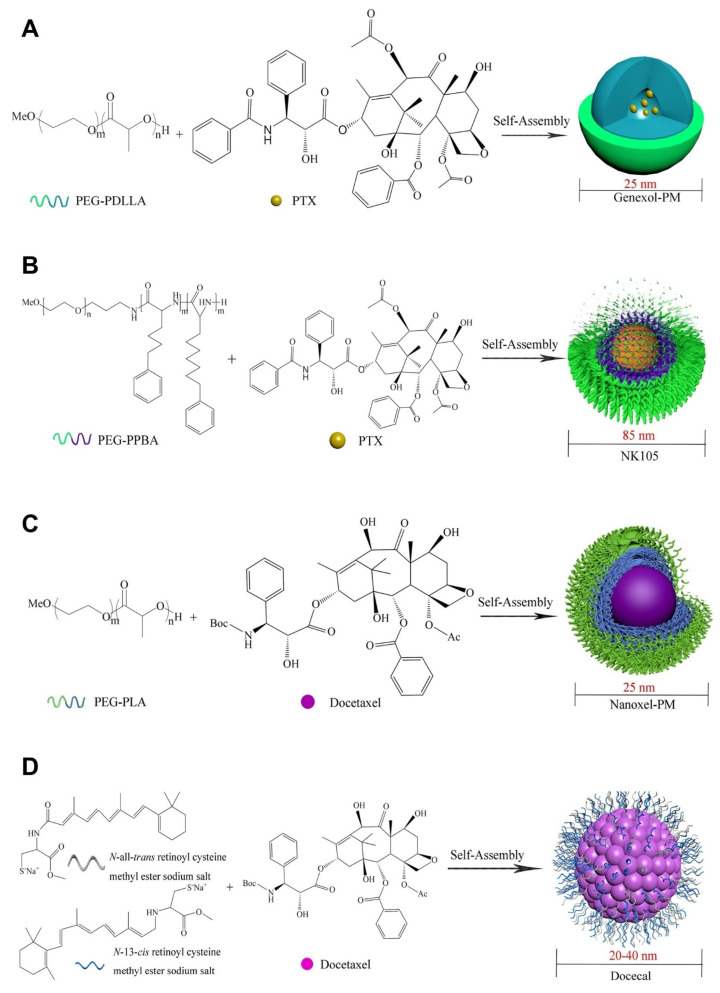

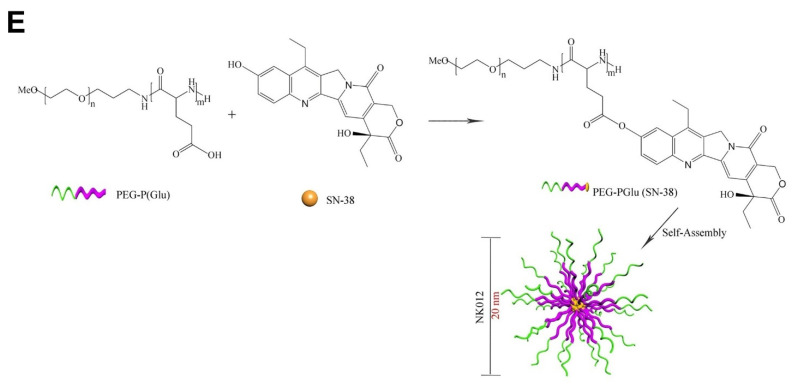
Chemical structures and schematic diagrams of PMs and respective drug candidates in the clinical trials, polymers used in the preparation (subscript represents molecular weights in Daltons) and drug molecule names are presented: (**A**) Genexol-PM block copolymer of poly(ethylene glycol)_2000_-*b*-poly(D,L-lactide)_1750_ and paclitaxel (PTX); (**B**) NK105 block copolymer of PEG_12000_-*b*-poly (4-phenyl-1-butanoate-L-aspartamide)_8000_ and paclitaxel (PTX); (**C**) Nanoxel-PM block copolymer of poly(ethylene glycol)_2000_-*b*-poly(Lactide)_1765_ and docetaxel; (**D**) Docecal XR-17 (N-all-trans retinoyl cysteine methyl ester sodium salt and N-13-cis retinoyl cysteine methyl ester sodium salt) and docetaxel; (**E**) NK012 block copolymer of poly(ethylene glycol)_12000_-*b*-poly(l-glutamic acid) and docetaxel modified and reprinted from [104] with permission from Elsevier, © 2022 Published by Elsevier B.V. on behalf of the Chinese Chemical Society and Institute of Materia Medica, Chinese Academy of Medical Sciences.

Metal drugs such as cisplatin and oxaliplatin have also been formulated in nanoforms such as NC-6004 and NC-4016, which are used in clinical trials. NC-6004 is a PEG-b-poly(glutamate) micelle for cisplatin that did not show any significant nephrotoxicity, indicating good safety and cytotoxicity profiles against tumor cells, whereas oxaliplatin-loaded PEG-b-P(Glu) block copolymer micelles (NC-4016) also showed good antitumor efficacy [108]. PM with DOX was developed as NK911, where DOX is covalently conjugated with PEG-b-P(Asp) and mainly developed for metastatic pancreatic cancer [109]. The DOX-loaded micelle SP1049C, whose activity has also been researched in breast cancer, was clinically approved against gastrointestinal cancer [110]. Another PM formulation, NC-6300 (NanoCarrier Co., Ltd., Chiba, Japan), has been developed for treating metastatic cancers. Here, the PM system was prepared by covalently conjugating EPB with the polymeric system of block copolymer PEG-polyaspartate (PEG-b-P(Asp)) with an acid-labile hydrazone bond [111]. A comprehensive summary of the structures and drug molecules of clinical trials is provided in Figure 7.

## 5. Regulatory Aspects

**Figure 8:**
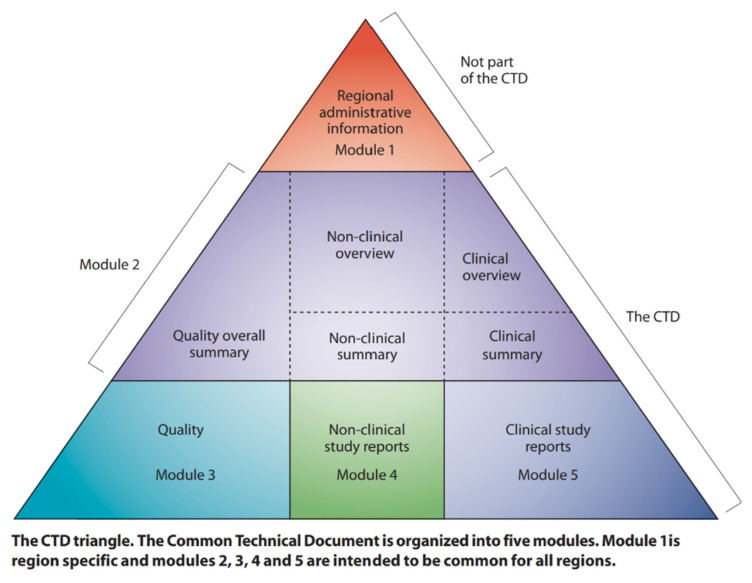
The Common Technical Document (Reference: [114]). The eCTD has five modules: 1. Administrative information and prescribing information. This is a country specific regional module, i.e., different for each region or country; 2. Common technical document summaries. This is a common module in all regions; 3. Quality; 4. Nonclinical study reports; 5. Clinical study reports. Image source: https://www.ich.org/page/ctd © International Council for Harmonisation of Technical Requirements for Pharmaceuticals for Human Use, https://www.ich.org/page/legal-mentions (accessed on 1 August 2022).

As seen through examples, very few nanobased formulations have been translated from the research setting to clinical trials, and very few have been approved for clinical use against various indications despite numerous studies about nanomedicines being available. The approval and rejection of such a formulation are heavily weighted and assessed by agencies formed by individual countries or regions such as the U.S. FDA, the European Medical Agency (EMA), the UK Medicines and Healthcare Products Regulatory Agency (MHRA), and the Japanese Pharmaceuticals and Medical Devices Agency (PMDA). The rules and assessment of nanotherapies may vary from agency to agency (see Figure 8).

### 5.1. Regulatory Guidelines, eCTD Submission Format in Various Countries

Advanced countries such as the USA, Canada, Japan, and the European Union follow the general guidelines as per ICH guidelines from 2017. More recently, China has started using eCTD documents for the drug registration process. India has its regulatory guidelines, however, largely based on the USFDA and ICH guidelines. A comprehensive guidance document is available on the USFDA website for guidance purposes (Figure 8) [113].

Majorly developed new nanotechnology fails the criteria for efficacy due to the insufficient amount of studies conducted to meet basic criteria for clinical trials, i.e., ease of fabrication, reproducibility, and long-term stability of the nanosystems. Hence, many of the reported formulations for cancer therapy have not advanced to an appropriate level [115]. Presently, major nanoparticles are liposome-based and are indicated for cancer. Therefore, a set of guidelines are available specifically for liposomal formulations or for commonly relatable nanoformulations. However, these guidelines hinder the entry of a new and different variety of nanoparticles that may have real potential. Nanotherapies present a variety of heterogeneous drug formulations. Each new nanoparticle technology results in different sizes, surface properties, morphology, and other attributes affecting in vivo drug behavior in comparison to traditional drug formulations. Therefore, evaluation and decision making by regulatory bodies to assess nanotechnology rely heavily on extensive data on quality, potency, and safety, along with the risk-benefit assessment, which becomes more complex and involves a considerable investment in time [116]. To speed up the assessment of NPs, regulatory authorities can rely on the database of nanoparticles. Similar to gene databases, data-based nanoformulations can be adopted worldwide and can show information about nanoparticles related to physical characterization, in vitro studies, in vivo results, and clinical-stage observation.

### 5.2. Guidelines Specific to PMs and Injectable Nanomedicines:

A properly designed nanomaterial database with up-to-date information should be designed to mine necessary information and assign nanostructure-based annotations. This knowledge helps facilitate the development of nanoparticles with definitive or near-predicted properties under biological conditions. With such a vision, the Nanoinformatics Roadmap 2030 was undertaken by the USA and the European Union, including a few other countries, in 2018 [117]. This initiative set the goal of integrating all the nanomaterial information by storing and sharing data. This initiative comprises the audience of academics and regulators and industry, helping to develop a common consensus about nanomaterials along with any health risks. Therefore, following this strategy, all the latest information regarding nanomaterials will be available in a single platform, aiding the growing research and development of strict policies for nanomaterials and facilitating their clinical translation.

The quality target product profile is derived by considering aspects such as micelle formulation, sterility, and pharmaceutical quality. The targets for the attributes were fixed based on the product label as desired by the respective agencies (examples are provided in Table 4).

The unit operations and the process parameters in the manufacturing process of micelle injections are very important and dependent on the manufacturing process. It is essential to develop scalable and controllable methods to achieve consistent results in the production process. Example unit operations and process parameters are provided for PMs in Table 5.

## 6. Conclusions and Future Perspectives

PMs represent a major contribution to the nanomedicine field, especially in anticancer therapy. PMs are next-generation drug delivery systems in the treatment of breast cancer and TNBC for delivering target-specific, poorly water-soluble drugs. PMs are known to enhance the loading efficiency of drugs and are easy to prepare in comparison to other nanoformulations. However, PM clinical translation is hindered primarily by nonpredictable stability in biological conditions and a lack of a cost-effective reproducible production strategy. Hence, the principal focus lies in overcoming these drawbacks. Thus, understating the relationship between the physicochemical properties and in vivo behavior of PM is an important area of investigation. This can be supported through extensive studies to determine the stability of formulation in various conditions such as in vitro and in vivo conditions such as oral administration, blood, saliva, etc. Furthermore, scalability could be improved by adopting technologies such as microfluidic technology [118].

**Figure 9:**
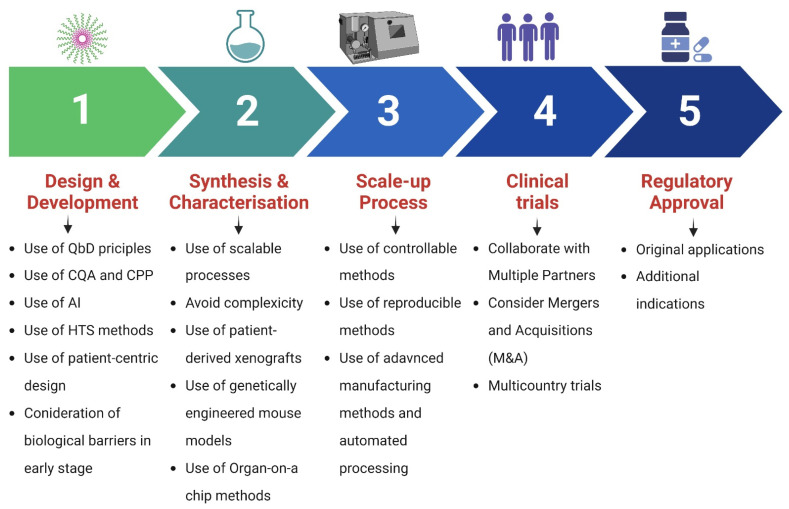
Steps and principles involved in the successful clinical translation of polymeric micelles. QbD, Quality by Design; CQAs, critical quality attributes; CPPs, critical process parameters; AI, artificial intelligence; HTS, high-throughput screening.

Moreover, the clinical translation of nanomedicines can be improved with the help of recent advances in science and technology. The emergence of machine learning and artificial intelligence has provided novel techniques for toxicity testing and can be employed for predicting nanoformulation toxicity. Such technology can be used to predict the biological response, pharmacokinetics, and behavior in the target organ [119]. In addition, nanomedicines should be chemically defined for both scientific and regulatory reasons, which will facilitate reproducibility and allow for the further iteration and elucidation of the underlying science. Collectively, the present limitations highlight the need to develop comparatively safe, simple, and lucrative nanomaterials for rapid translation in clinical therapeutics in the treatment of different types of breast cancer. To accelerate the clinical translation of PMs, it is important to focus on the design, synthesis, characterization, and scale-up processes from the beginning to overcome hurdles in the clinical trials and regulatory approval stage. Figure 9 summarizes the steps and principles involved in the successful translational outcome.

## Figures and Tables

**Table 1:** Characterization techniques used in measuring various PM properties.

S.No.	Characterization Method	Property Measured	Reference
1	NMR	Nature of amphiphilic components	[42,43]
2	FTIR	Nature of amphiphilic components	[44]
3	DLS	PM size	[45,46]
4	AFM	PM morphology	[47]
5	Cryo-TEM	PM morphology	[48]
6	Surface tension	CMC of PM	[49]
7	Absorbance (Sudan III, 1,6-diphenyl-1,3,5-hexatriene (DPH))	CMC of PM	[50]
8	Fluorescence (Pyrene, Coumarin-6, Nile Red, 1,6-diphenyl-1,3,5-hexatriene (DPH))	CMC of PM	[51]
9	DLS	Surface charge of PM	[14,15]
10	FRET	Drug release, Protein corona formation, Stability in biological fluids	[52]
11	Chromatography Techniques	Drug release, Protein corona formation, Stability in biological fluids, Tissue concentrations	[14,15]
12	Symmetrical flow field-flow fractionation (AF4)	Stability in biological fluids (serum, cerebrospinal fluid, vitreous (eye))	[14,15]
13	X-rays	PM size	[53]

**Table 2:** List of the different types of PMs developed against breast cancer.

S.No.	Polymer	Therapeutic Agent	Disease Type	Ref.
1	PLA-PEG	LAP, PTX	HER-2-negative, HER-2-positive	[67]
2	P(MeOx-*b*-BuOx-*b*-MeOx	PTX, alkylated cisplatin	Multidrug-resistant breast cancer	[68]
3	PEG-G3-PEITC	PTX	Breast cancer	[69]
4	Nonionic pluronic block copolymers	DOX	TNBC	[70]
5	PEG-PGlu-PPhA	DOX, ALN	Late-stage breast cancer bone metastasis	[71]
6	Zein with lactoferrin protein	RAP, WOG	Breast cancer	[72]
7	MPEG-PCL	GA	Breast cancer	[73]
8	Pluronic F127 block copolymer and Vitamin E-TPGS	RES	Breast cancer	[74]
9	Pluronic F127 block copolymer	Zileuton	TNBC	[75]
10	POEGMEMA-PtBuMA	Estrogen	Hormone-responsive breast cancer	[76]
11	PF-FA	FS	Breast cancer	[77]
12	INT	DOX, PTX	Breast cancer	[78]
13	POEG-VBC	DOX, tRNA-mir-34a	Metastatic breast cancer	[79]
14	Pluronic PF127-pluronic F68 with folate	CH	Breast cancer	[80]
15	Pluronic F127 with HA-DOCA-His-PF	DOX	Multidrug-resistant breast cancer	[81]
16	Cholic acid with poly (bis (carboxyphenoxy) phosphazene	PTX	Breast cancer	[82]
17	BD-PEI and BD-peptide-PEG	DTX	Metastatic breast cancer	[83]
18	PEG-poly(amino acid) block	EPI	ALNM of TNBC	[84]
19	PEOz-PLA	PTX, HNK	Multidrug-resistant and metastatic breast cancer	[85]
20	PELA-PBAE	PTX	Metastatic breast cancer	[86]
21	mPEG-CS-Hz-CH	PTX	Breast cancer	[87]
22	PLGA-BS-ATRAM	DOX-TPP	Breast cancer	[89]
23	mPEG-Dlabile-PCL-Fc	DOX	Breast cancer	[90]
24	mPEG-PCL	MTX	Breast cancer	[60]
25	mPEG-b-poly(εCL-co-αN3εCL	MTX	Breast cancer	[91]
26	HA-ss-BF	DOX	Breast cancer	[92]
27	PEG-PEI-PCL-S–S-PCL-PEG (PPPT)	DOX	Breast cancer	[93]
28	PEG, PPE	DOX	Multidrug-resistant breast cancer	[64]
29	PEG, polycaprolactone, RGD peptide	DOX, ICG	Breast cancer	[94]
30	poly(Tyrosine(alkynyl)-OCA), monomethoxy poly(ethylene glycol)-b-poly(Tyrosine(alkynyl)-OCA)	Camptothecin	Breast cancer	[95]
31	P_5k_SSLV	DOX	Multidrug-resistant breast cancer	[96]
32	DSPE-PEG2000	IR780	Breast cancer lymphatic metastasis	[97]
33	mPEG-PCL copolymer	DTX, IR780	Breast cancer	[98]
34	Poly(N-isopropylacrymaide) and polylactic acid	Shikonin	Breast cancer	[99]
35	Zein-lactoferrin copolymer, Fe_3_O_4_ nanoparticles	DAS	TNBC	[52]
36	Folate-conjugated dextran/retinoic acid copolymer, oleic acid	DOX	Breast cancer	[65]
37	mPEG-b-PHEP copolymer, iron oxide nanocubes	Emodin	Breast cancer	[66]
38	CSO-SS-Hex, HA, Speg copolymer, Fe_3_O_4_ nanoparticles	GA	TNBC	[100]
39	SHR, PLGLAGK-Mal, coated with anti-CD28 antibody	CH223191	Breast cancer	[101]
40	HPMA, OEGMA, GFLG peptide	DOX, Nifuroxazide	Metastatic breast cancer	[102]

**Table 3:** PMs that are clinically approved and undergoing clinical trials [112].

S.No.	PM	Excipient	Drug Release	Therapeutic Agent	Disease Type	NCT Number/ Clinical Phase (Status)
1	Genexol-PM	PEG-b-PLA	Controlled release with no specific triggering mechanism	PTX	Breast Cancer	NCT00876486/Phase III (Completed)
				PTX, PM with DOX	Metastatic Breast Cancer	NCT01784120/Phase II (Unknown)
				PTX	Recurrent Breast Cancer	NCT00912639/Phase IV (Unknown)
				PTX	Breast Cancer	NCT01169870/Phase II (Withdrawn)
				PTX, PM with Carboplatin	Ovarian Cancer	NCT00877253/ Phase I (Completed)
2	NK105	PEG-b-PPBA	Sustained release equivalent dose of <1 μg mL^−1^ with no specific triggering mechanism	PTX	Metastatic or Recurrent Breast Cancer	NCT01644890/ Phase III (Completed)
3	Nanoxel-PM	PEG-PDLLA	DTX was released immediately from micelle to bind to the plasma proteins. Pharmacokinetics equivalent to Taxotere^®^	DTX	Breast Cancer, Non-small Cell Lung Cancer	NCT04066335/(Recruiting)
			No specific triggering mechanism	PTX	Advanced Breast Cancer	NCT00915369/ Phase I(Unknown)
4	Docecal	XR-17 block polymer	Controlled release with no specific triggering mechanism	DTX	Breast Cancer	2012-005161-12 (EudraCT)/Phase III (results not published)
5	NK012	PEG-b-poly(glutamate)	Releases SN-38 under neutral conditions even without the presence of a hydrolytic enzyme	SN-38 an active metabolite of irinotecan hydrochloride (CPT-11)	Triple Negative Breast Cancer	NCT00951054/Phase II/Completed
			Releases SN-38 under neutral conditions	NK012 and Carboplatin	Metastatic Triple Negative Breast Cancer	NCT01238952/Phase 1/Completed

**Table 4:** Representative critical quality attributes (CQA) for the polymeric micellar injectable dosage form.

S.No.	Quality Attribute	Target Terms	Criticality	Justification
1	Description	Examples: A sterile, translucent red/green/yellow micellar dispersion	Yes	✓ Critical for safety and efficacy. ✓ Controlled by the quality management system.
2	pH	Target pH	Yes	✓ Critical for safety and efficacy. ✓ The limits were fixed based on the available information.
3	Osmolality (mOsm/kg)	Specific mOsm/kg of water	Yes	✓ Osmolality is having a direct effect on the safety and efficacy of the product. ✓ The range for osmolality is fixed based on the analysis. ✓ An infusion after dilution may affect this parameter.
4	Average particle size	Particle size in nanometers (nm)	Yes	✓ Particle size has a direct impact on the safety and efficacy of the formulation. ✓ Micelles larger in size than the gaps between endothelial cells lining the tumor blood vessels will not be able to extravasate into the tumor tissue which determines product efficacy. ✓ Conversely, if micelles are too small, then they are not stable, which may affect patient safety. ✓ The average particle size is dependent on the synthetic step.
5	Particle size distribution	Particle size in nanometers (nm) Population distribution (D,10), (D,50), (D,90)	Yes	✓ Particle size distribution may have an impact on the safety and efficacy of the formulation. ✓ The limits of D10, D50, and D90 will be defined.
6	Identification (API)	Positive for specific drug substance	Yes	✓ Critical for safety and efficacy, this CQA can be effectively controlled by the quality management system.
7	Identification of polymers	Positive for specific polymers (PLGA, PLA, etc.)	Yes	✓ Critical for safety and efficacy, this CQA can be effectively controlled by the quality management system.
8	Assay (Dug substance)	Each mL contains a specific amount of labeled drug	Yes	✓ Variability in the assay will affect safety and efficacy. Therefore, it is critical.
9	Polymer content	Each mL contains the amount of polymer	Yes	✓ Polymer content directly affects the safety and efficacy of the formulation. ✓ Content of individual polymers determines the in vivo stability, prolonged blood circulation, drug retention, and its release, which affects the efficacy of the product and patient safety.
10	Percentage of the entrapped drug	Should comply with the qualified limits. Example: Not less than 95.0% of the assay amount.	Yes	✓ Percentage entrapped and unentrapped may have an impact on the safety and efficacy of the formulation. ✓ The rationale behind encapsulation of drug into micelles is for site-specific tumor targeting. ✓ In addition, the presence of more of the free drug would lead to an increase in the probability of toxicity. Hence, it is considered critical.
11	Percentage of Unentrapped drug	Should comply with the qualified limits Example: Not more than 5.0%.	Yes	
12	Ion content (ppm)	Total Ion content (ppm) (Ammonium, Iron, etc.).	Yes	✓ Any unwanted ions will affect patient safety.
14	Related substances (API)	Should comply with the qualified limits.	Yes	✓ Related substances of the API and the polymers added in the formulation may affect the safety and efficacy of the product. Hence, it is critical.
15	Related substances (polymers)	Should comply with the qualified limits.	Yes	
16	Drug release	Drug release profile.	Yes	✓ The drug release profile is important for bioavailability (BA); therefore, it is critical.
17	Visible Particulate Matter Particulate Matter (sub visible)–Microscopic method	Free from visible particles. Not more than 3000 average number of particles should be greater than or equal to 10 µm. Not more than 300 average number of particles should be greater than or equal to 25 µm.	Yes	✓ Important for IV infusions. ✓ The absence of particulate matter is of paramount importance to ensure patient safety. Hence, it is considered critical. However, it is controlled by the quality management system.
18	Uniformity of dosage units	Content uniformity method.	Yes	✓ Variability in uniformity of dosage units will affect safety and efficacy. Hence, it is considered critical.
20	Zeta Potential (by Zeta Sizer)	Defined by innovator.	Yes	✓ Extremes in zeta potential values would lead to repulsion or aggregation and hence will affect the stability. Hence, it is considered critical.
21	Sterility	The product should be sterile.	Yes	✓ To ensure the safety of patients, the product should be sterile. Hence, it is considered critical.
22	Bacterial Endotoxin Test	Specific limits.	Yes	✓ To ensure the safety of patients, bacterial endotoxin in the product should be low. Hence, it is considered critical.
23	Residual solvents	Based on process optimization.	Yes	✓ Excess amounts of solvents in the product may affect the quality.

**Table 5:** Unit operations and the critical process parameters for the preparation of PMs.

S.No.	Unit Operation	Process Parameter
1	Polymer dissolution	Process Temperature
		Stirring speed
		Stirring Time
2	Preparation of micelles	Process Temperature
		Homogenization Speed
		Homogenization Time
3	Extrusion process	Process Temperature
		Number of Cycles
		Membrane Pore Size
		Pump Speed
4	Dia-filtration (Removal of ions)	Conductivity of Filtrate
		Process Temperature
		Pump Speed
5	Drug Loading	Process Temperature
		Stirring Speed
		Incubation Time
6	Filtration	Filter Flushes
		Filter Integrity
		Filtration Time
		Filtration Pressure

## Data Availability

The authors declare that the review article materials of this study are available within the article.
